# Splenic hydatid disease in pregnancy

**DOI:** 10.4102/sajhivmed.v23i1.1363

**Published:** 2022-05-26

**Authors:** Kirstie F. Thomson, Florence Mahlobo, Denasha L. Reddy

**Affiliations:** 1 Department of Internal Medicine, Faculty of Health Sciences, University of the Witwatersrand, Johannesburg, South Africa; 2 Department of Radiology, Faculty of Health Sciences, University of the Witwatersrand, Johannesburg, South Africa; 3 Department of Medical Imaging, Faculty of Health Sciences, University of Johannesburg, Johannesburg, South Africa

**Keywords:** cystic echinococcosis, hydatid disease, pregnancy, spleen, splenomegaly

## Abstract

**Introduction:**

Hydatid disease in the South African setting remains an important differential diagnosis in many appropriate clinical presentations, such as splenomegaly. Splenic hydatid disease in pregnancy is a rare and complex disease to manage.

**Patient presentation:**

In this case report we describe a case of isolated splenic hydatid disease in an HIV-positive woman presenting in her third trimester of pregnancy.

**Management and outcome:**

A multidisciplinary team consisting of specialists from the high-risk maternity unit, hepatobiliary surgery and infectious diseases planned the management of the patient, which included pre-operative albendazole and elective caesarean section with assisted forceps delivery at 36 weeks’ gestation. An elective splenectomy in the post-partum period was planned for definitive management.

**Conclusion:**

Our aim is to highlight the unique treatment challenges of hydatid disease in pregnancy and the need for a multidisciplinary team approach when managing complex cases of hydatid disease.

## Introduction

Hydatid disease is a parasitic infestation by a dog tapeworm of the genus Echinococcus.^1^ Cystic echinococcosis is the larval cystic stage of *Echinococcus granulosus* and may cause illness in intermediary hosts such as sheep and occasionally humans.^1^ In the typical dog-sheep lifecycle, the adult tapeworms inhabit the small intestines of definitive carnivorous hosts such as dogs and the eggs passed in the faeces may be ingested by a grazing sheep.^1^ Humans play the same role as these intermediary hosts and can be infected by ingesting food or drink contaminated with faecal material containing tapeworm eggs.^1^ The clinical features of cystic echinococcosis are highly variable and the wide spectrum of disease presentation differs according to various factors, such as the involvement of a particular organ, the nature and size of the cysts as well as their effect on surrounding structures.^1^ Complications relating to these cysts are not uncommon and include ruptured cysts, bacteriological infection or immunological reaction to the cysts themselves.^1^ Diagnosis is usually made using a combination of serological tests and imaging.^1,2^ Although isolated splenic hydatid disease is a rare clinical entity with a prevalence of between 2.5% and 5.8% of all patients with hydatid disease, it is the third most commonly affected organ after the liver and lungs.^1,2^ While the exact pathophysiology for the involvement of the spleen is largely unknown, there are a few mechanisms that could explain the splenic disease process. The parasitic eggs can penetrate the barrier of the liver and the lungs, thus entering the systemic circulation and enter the spleen where they may settle.^2^ Additionally, there is the possibility of retrograde spread via the portal vein, colonic trans-parietal passage of the eggs or intraperitoneal rupture of a hydatid cyst with subsequent ‘seeding’.^2^ The incidence of hydatid disease in pregnancy is largely unknown, and management in this clinical scenario is determined on a case-by-case basis. According to recent literature, there is no conclusive data to suggest that HIV co-infection is a risk factor for developing hydatidosis. However, a recent prospective study in a South African setting suggested that HIV co-infected patients more often had infected cysts and required emergency surgery more frequently for hepatic hydatid disease than their HIV-negative counterparts.^3^ Comparative studies showed that there seems to be an impaired immune response to *E. granulosus* in patients with HIV and hydatid disease severity might be offset by viral suppression using antiretroviral therapy in HIV infected patients.^4^

## Case presentation

**FIGURE 1:**
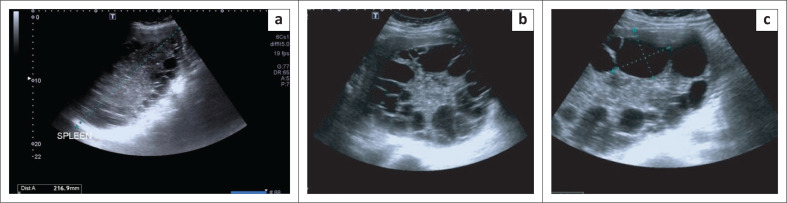
Images taken from the initial ultrasound done on presentation; (a) An image showing the enlarged spleen measuring 21 cm; (b) This image shows the extensive splenic hydatid cysts involving more than 90% of the normal splenic tissue; (c) An indication of the largest splenic cyst measuring 4.8 cm × 4.0 cm.

A 41-year-old pregnant woman at 35 weeks gestation by early dates presented to our tertiary infectious diseases clinic with a 7-month history of progressively worsening left flank fullness, associated left shoulder pain and clinical findings of a massive splenomegaly. A formal ultrasound confirmed a notably enlarged spleen measuring 21 cm and multiple splenic cystic lesions of varying size, with the largest measuring 4.8 cm × 4 cm (Figures 1a and 1c). The cysts involved more than 90% of the normal splenic tissue (Figure 1b). The liver was normal and a single intra-uterine pregnancy was confirmed at this time. There was no suspected foetal intra-uterine growth restriction according to the obstetric team during the multidisciplinary team meeting and the gestational age of the foetus was confirmed.

These ultrasound findings were suggestive of splenic hydatid disease. An indirect haemagglutinin assay (IHA) for echinococcosis was strongly positive with a titre of 1 in 1024.

The patient was diagnosed with HIV eight years ago and was currently virologically suppressed with a CD4 cell count of 500 cells/uL on first-line antiretroviral therapy (tenofovir, emtricitabine and efavirenz).^5^ She had been successfully treated for tuberculous arthritis eight years ago and, according to the patient, has been adherent to antiretroviral therapy since diagnosis. Obstetric history revealed four full-term pregnancies and one first trimester miscarriage. The first two pregnancies were normal vaginal deliveries and the latter two were caesarean sections due to cephalopelvic disproportion. The patient was otherwise well and had no previous hospital admissions, treatment of parasitic infections or anaphylactic reactions and, to her knowledge, this was her first diagnosis of hydatid disease. She grew up in a rural village in the Eastern Cape, but resided in Katlehong, Gauteng.

A multidisciplinary team, including specialists from the high-risk maternity unit, hepatobiliary surgery and infectious diseases was quickly formed to plan the patient’s further management. On discussion with obstetricians at the high-risk maternal clinic, it was decided that it was safer to deliver the patient at 36 weeks as opposed to later on so as to avoid spontaneous labour (uterine contractions) as far as possible, in order to prevent cyst rupture due to the sheer size of the cysts. The compressive nature of the splenic hydatid disease was already causing subjectively worsening symptoms for the patient as her pregnancy progressed. The delivery at 36 weeks allowed the patient to receive five days of oral albendazole and a planned caesarean section could take place with assisted forceps delivery in order to avoid fundal pressure as much as possible. The foetus was of adequate size according to late ultrasound and showed no signs of intra-uterine growth restriction and thus every possible measure was made to achieve a balance between avoiding cyst rupture and ensuring safety of the foetus.

There were no complications peri-operatively and a healthy infant was delivered. Following the procedure, the patient continued albendazole 400 mg orally twice daily for six weeks. The discharge plan included routine pre-splenectomy vaccinations and elective splenectomy. The patient was lost to follow-up due to financial reasons, but re-presented and is currently awaiting elective splenectomy.

## Discussion

Regarding management of splenic hydatid disease, a multimodal approach is required and definitive surgery, by way of splenectomy, is considered the gold standard.^6^ This intervention however is controversial during pregnancy and the relative timing remains difficult due to the possible precipitation of preterm labour and abortion.

The three treatment options available for splenic hydatid disease are: puncture, aspiration, injection of protoscolicidal agent and re-aspiration (PAIR); oral antiparasitic therapy (using either albendazole alone, or in combination with praziquantel) or surgery (total splenectomy or spleen-conserving treatment).^6^

A review showed that, of these approaches, PAIR was used the least although this approach could be most beneficial in patients with cysts of a relatively small diameter (< 50 mm), patients with splenic hydatid cysts of type I or II Gharbi ultrasound classification or patients who refuse surgery or pose a high anaesthetic risk.^6^ Of note, albendazole was used in conjunction with the PAIR approach in order to reduce cyst size and decrease subsequent risk of an anaphylactic reaction and recurrence.^6,7^ The use of pharmacological therapy as a primary treatment modality in splenic hydatid disease is discouraged.^6^

Total splenectomy is preferred when more than 75% of the spleen is involved, when multiple cysts occur or when there are centrally located cysts.^6^

Despite the vast number of publications on echinococcosis, there remain only a few cases related to pregnancy and the incidence is thought to be as low as 1 in 20 000–30 000.^8^ This lack of data has resulted in the lack of consensus on an internationally standardised approach to treatment.^9^ Even more obvious is the lack of publications relating to splenic hydatid disease in pregnancy as most of the cases described involve the liver or lungs. A case report from Israel described the management of isolated splenic hydatid disease in pregnancy; however the patient was at five weeks’ gestation and management included termination of the pregnancy on presentation.^10^

It is important to note that due to the decreased cell-mediated immunity during pregnancy, there may be a rapid increase in parasitic growth and size of the cysts leading to critical complications such as rupture, compression of adjacent structures or communication with the biliary tree when hepatic hydatid disease is present.^9,11,12^. The enlarging uterus may cause pressure on the cysts, increasing the risk of cyst rupture, inevitable dissemination and possible spillage into the peritoneal cavity and subsequent anaphylaxis.^9,12^

Hydatid disease in pregnancy may result in significant maternal morbidity and possible mortality, as described by Robertson et al. in a case of a potentially preventable maternal death in South Africa, due to complications of hydatid cysts rupture.^13^

Surgical intervention of the cysts during the latter stages of pregnancy carries a greater chance of cyst rupture and may potentially precipitate early onset labour.^9^

Due to the limited data available in managing splenic hydatid disease in pregnancy, experts suggest a case-by-case assessment.^7,9^ Albendazole should be avoided in the first trimester due to potential teratogenic effects and all invasive therapeutic interventions that are deemed necessary should ideally be performed at 20–24 weeks gestation.^10^ Anti-helminthic drugs are considered safe in the latter stages of pregnancy; however, in the published studies where albendazole had been used in obstetric patients, the reported doses used were far lower than those required in hydatid disease.^13^ Medical management alone may be required in certain instances where the patient refuses surgical intervention, such as the case described by Tyagi et al. where albendazole was shown to reduce the size of daughter cysts although it had a limited affect in cyst reduction where the cysts were large.^14^ Despite a number of successful case reports, there is currently no data available to support medical treatment alone for hydatid disease in pregnancy.^13^

Hydatid disease has a post-surgical recurrence rate of about 10%; however, this risk is increased when the surgical procedure is performed in pregnancy.^7^ This is thought to be due to the decrease in cell-mediated immunity in the gravid state.^7^ Peritoneal recurrence can even present 4–15 years after splenectomy.^15^

The potential use of exogenous steroids has been explored in hydatid disease. This would be especially relevant if steroids were indicated in possible preterm labour, prior to foetal lung maturity. A case by Diem et al. demonstrated the harmful effect of steroids in hydatid disease in a patient where alveolar Echinococcus disease was accelerated after the patient was given steroids for autoimmune encephalitis.^16^ The evidence is, however, limited, as only alveolar involvement was discussed. It was also suggested by Romano et al. that the growth, differentiation and performance of certain parasitic species is enhanced by corticosteroids; however, this observation was based on in vitro studies and has not yet been demonstrated clinically in patients with splenic hydatid disease.^17^ However, observational studies have suggested the benefit of using peri-operative steroids to ameliorate potential anaphylactic reactions from cyst rupture in cases where surgery is the definitive treatment option.^18,19^

Although there is possible benefit from using steroids during the peri-operative phase of treatment, there is still potential for harm and there is currently no conclusive evidence to support their use in hydatid disease during pregnancy. The use of steroids should be considered on a case-by-case basis.

The prevention of hydatid disease in endemic areas could be achieved by education regarding the modes of transmission of the disease and simple hygiene techniques when working with sheep viscera and in areas where dogs frequent.^1^ The stage of the disease was advanced in our patient and highlighted the indolent nature of the disease process. Therefore, emphasis needs to be placed on prevention of infection in endemic areas at an early age, rather than targeting prevention in pregnant women. Symptoms of pregnancy such as nausea, vomiting and abdominal discomfort may mimic abdominopelvic hydatid disease and therefore a high index of suspicion, especially in an endemic area like South Africa, is required.

In our case review, due to the impending rupture of the cysts and the isolated splenic involvement, we believed a multi-step approach would be best for adequate management. Delivery via elective caesarean section with assisted forceps at 36 weeks’ gestational age, avoided, as far as possible, spontaneous uterine contractions with further pressure-related complications on the cysts. Due to > 90% of the spleen being involved, a total splenectomy was deemed safer than other surgical techniques. Albendazole was utilised as an adjunctive pharmacologic therapy in order to prevent recurrence of the disease.

## Conclusion

Hydatid disease in pregnancy may result in significant maternal morbidity and possible mortality due to complications of hydatid cyst rupture.^13^ There remains no standardised guideline for the management of hydatid disease in pregnancy and therefore the management of each case should be individualised. A multidisciplinary team was formed early due to the immediate concern for complications in both the mother and the foetus. The late presentation to a specialised facility left limited time for decision-making. Our aim in describing this patient’s unique presentation and subsequent successful management of her delivery is to contribute to the knowledge base of managing splenic hydatidosis in late pregnancy.

Clinicians managing cases of hydatid disease in specific patient populations, such as pregnancy, should be encouraged to document them in order to contribute to knowledge in this important and neglected area.

## References

[1] Brunetti E, Filice C. Echinococcosis Hydatid cyst [homepage on the Internet]. emedicine.medscape.com; 2021 [cited 2020 Jan 24]. Available from: https://emedicine.medscape.com

[2] Milosavljevic V, Veselinovic M, Tadic B, et al. Laparoscopic management of initially unrecognized splenic hydatid cysts: A case report and review of the literature. Medicina. 2019;55(12):771. 10.3390/medicina55120771 PMC6956320 31817008

[3] Kloppers C, Couzens-Bohlin K, Bernon M, et al. Hydatid disease in South-Africa – Is it a different disease in patients with HIV co-infection?. HPB. 2019;21(Suppl 3):S593. 10.1016/j.hpb.2019.10.235

[4] Bohlin K, Kotze U, Lindemann J, et al. Impact of HIV co-infection in patients presenting with hepatic hydatid disease. HPB. 2020;22(Suppl 2):S256. 10.1016/j.hpb.2020.04.140

[5] National Department of Health: South Africa. 2019 ART clinical guidelines for the management of HIV in adults, pregnancy, adolescents, children, infants and neonates [homepage on the Internet]. National Institute of Communicable Disease; 2021 [cited 2020 Jan 20]. Available from: https://www.nicd.ac.za/wp-content/uploads/2019/11/2019-ART-Clinical-Guidelines-25-Nov.pdf

[6] Akbulut S, Sogutcu N, Eris C. Hydatid disease of the spleen: Single-center experience and a brief literature review. J Gastroenterol. 2013;17(10):1784–1795. 10.1007/s11605-013-2303-5 23949423

[7] Bhattacharyya S, Bhattacharya S, Alam H, Patua B, Chattopadhyay P. Dilemmas encountered while dealing a pregnancy complicated by pelvic Hydatid disease. Arch Gynecol Obstets. 2013;288(5):965–966. 10.1007/s11605-013-2303-5 23625356

[8] Can D, Oztekin O, Oztekin O, Tinar S, Sanci M. Hepatic and splenic hydatid cyst during pregnancy: A case report. Arch Gynecol Obstets. 2003;268(3):239–240. 10.1007/s00404-002-0348-x 12942259

[9] Rodrigues G, Seetharam P. Management of hydatid disease (echinococcosis) in pregnancy. Obstet Gynecol Surv. 2008;63(2):116–123. 10.1097/OGX.0b013e3181601766 18199385

[10] Saher SF, Sayfan J. Echinococcosis of the Spleen during pregnancy. IMAJ. 2006;3:290–291. 11344847

[11] Celik S, Okyay O, Karaman E, Sert OZ, Clm N, Okyay T. Analysis of factors affecting outcomes of pregnancy complicated by Echinococcus: an algorithm for approach and management. Arch Gynecol Obstets 2018;1:103–110. 10.1007/s00404-018-4792-7 29785547

[12] Sahin E, Nayki U, Sadik S. Abdominal and pelvic hydatid disease during pregnancy. Arch Gynecol Obstet. 2005; 273:58–59. 10.1007/s00404-004-0679-x 16200401

[13] Robertson M, Geerts L, Gebhardt G. A case of hydatid cyst associated with postpartum maternal death. Ultrasound Obstet Gynecol. 2006;27(6):693–696. 10.1002/uog.2763 16628613

[14] Tyagi S, Singh C, Tripathi R, Mala Y. Pregnancy complicated by abdominopelvic hydatid disease. BMJ Case Rep. 2012;2012:bcr2012007880. 10.1136/bcr-2012-007880 PMC4545115 23230263

[15] Rodrigues-Leal GA, Moran-Villota S, Milke-Garcia LNM. Splenic hydatidosis: A rare differential diagnosis in a cystic lesion of the spleen. Rev Gastroenterol Mex. 2007;72:122–125. 17966372

[16] Diem S, Gottstein B, Beldi G, Semmo N, Diem L. Accelerated course of alveolar echinococcosis after treatment with steroids in a patient with autoimmune encephalitis. Cureus. 2021;13(10):e18831. 10.7759/cureus.18831 34820209 PMC8596569

[17] Romano M, Jiménez P, Miranda-Brito C, Valdez R. Parasites and steroid hormones: Corticosteroid and sex steroid synthesis, their role in the parasite physiology and development. Front Neurosci. 2015;9:224. 10.3389/fnins.2015.00224 26175665 PMC4484981

[18] Gupta R, Sharma S, Prabhakar G, Mathur P. Hydatid disease in children: Our experience. Formosan J Surg. 2014;47(6):211–220. 10.1016/j.fjs.2014.12.001

[19] Tercan M, Tanriverdi T, Kaya A, Altay N. Our clinical experience and follow-up results in hydatid cyst cases: A review of 393 patients from a single center. Braz J Anesthesiol. 2020;70(2):104–110. 10.1016/j.bjan.2019.12.013 32532549 PMC9621146

